# The relationship between the recognition of specific basic emotions and negative symptom domains in patients with schizophrenia spectrum disorders

**DOI:** 10.1192/j.eurpsy.2022.305

**Published:** 2022-09-01

**Authors:** M. Zierhut, K. Boege, N. Bergmann, I. Hahne, A. Braun, J. Kraft, T.M.T. Ta, S. Ripke, M. Bajbouj, E. Hahn

**Affiliations:** 1 Charité - Univerversitätsmedizin Berlin, Campus Benjamin Franklin, Department For Psychiatry And Psychotherapie, Berlin, Germany; 2 Berlin Institute of Health at Charité – Universitätsmedizin Berlin, Bih Academy, Clinician Scientist Program, Berlin, Germany; 3 Charité – Universitätsmedizin Berlin, Campus Mitte, Department Of Psychiatry And Psychotherapy, Berlin, Germany

**Keywords:** schizophrénia, Emotion recognition, negative symptoms, Psychosis

## Abstract

**Introduction:**

Current research suggests emotion recognition to be significantly impaired in individuals with schizophrenia spectrum disorders (SSD), whereby negative symptoms are theorised to play a crucial role. Emotion recognition deficits are assumed to be predictors of transition from clinical high risk to schizophrenia. So far, little attention has been given hereby to the subdomains of negative symptoms and recognizing the individual basic emotions.

**Objectives:**

Our study aimed to explore the relationship between the recognition of the basic emotions and each negative symptom domain.

**Methods:**

66 patients with a SSD diagnosis were recruited at the Charité – Universitätsmedizin Berlin. Correlational and regression analyses to control for the covariates (age, education, sex) were conducted between the recognition of the six basic emotions (anger, disgust, fear, happiness, sadness, surprise) using the Emotion Recognition Task of the Cambridge Neuropsychological Test Automated Battery (CANTAB) and the seven different subdomains of negative symptoms of the Positive and Negative Syndrome Scale (PANSS).

**Results:**

revealed significantly negative correlations of blunted affect with the recognition of happiness, fear, and disgust. Difficulties in abstract thinking, also correlated positively with the recognition of fear. Additionally, we found a significant positive correlation between stereotyped thinking and difficulties in abstract thinking with the response latency in emotion recognition.

**Conclusions:**

Individuals with SSD and domains of negative symptoms showed specific impairments in recognizing the representation of basic emotions. A longitudinal design to make causality statements would be useful for future research. Moreover, emotion recognition should be considered for early detection and individualized treatment.

**Disclosure:**

No significant relationships.

